# Actionable mutational profiling in solid tumors using hybrid‐capture‐based next‐generation sequencing in a real‐world setting in Spain

**DOI:** 10.1002/cam4.6827

**Published:** 2024-01-11

**Authors:** Sandra Zazo, Sandra Pérez‐Buira, Nerea Carvajal, Jenifer Plaza‐Sánchez, Rebeca Manso, Nuria Pérez‐González, Carolina Dominguez, Iván Prieto‐Potin, Jaime Rubio, Manuel Dómine, Virginia Lozano, Patricia Mohedano, David Carcedo, Rafael Carias, Federico Rojo

**Affiliations:** ^1^ Department of Pathology Fundación Jiménez Díaz University Hospital Madrid Spain; ^2^ IIS‐Fundación Jimenez Diaz Center for Biomedical Network Research on Cancer (CIBERONC) Madrid Spain; ^3^ Medical Oncology Department Fundación Jiménez Díaz University Hospital Madrid Spain; ^4^ Roche Farma S.A. Madrid Spain; ^5^ Hygeia Consulting SL Madrid Spain

**Keywords:** actionable mutation, lung cancer, molecular alterations, next‐generation sequencing, solid tumor

## Abstract

**Objective:**

This study aimed to describe the performance of a next‐generation sequencing (NGS) panel for the detection of precise genomic alterations in cancer in Spanish clinical practice. The impact of tumor characteristics was evaluated on informative NGS and actionable mutation rates.

**Materials and Methods:**

A cross‐sectional study was conducted at the Fundación Jiménez Díaz University Hospital (May 2021–March 2022) where molecular diagnostic of 537 Formalin‐Fixed Paraffin‐Embedded (FFPE) tissue samples of diverse solid tumors (lung, colorectal, melanoma, gastrointestinal stromal, among others) was performed using AVENIO Tumor Tissue Targeted Kit. A descriptive analysis of the features of all samples was carried out. Multivariable logistic analysis was conducted to assess the impact of sample characteristics on NGS performance defined by informative results rate (for all tumors and for lung tumors), and on actionable mutations rate (for lung tumors only).

**Results:**

AVENIO performance rate was 75.2% in all tumor samples and 75.3% in lung cancer samples, and the multivariable analysis showed that surgical specimens are most likely to provide informative results than diagnostic biopsies. Regarding the mutational findings, 727 pathogenic, likely pathogenic, or variant of unknown significance mutations were found in all tumor samples. Single nucleotide variant was the most common genomic alteration, both for all tumor samples (85.3% and 81.9% for all solid tumors and lung samples, respectively). In lung tumors, multivariable analysis showed that it is more likely to find actionable mutations from non‐smokers and patients with adenocarcinoma, large cell, or undifferentiated histologies.

**Conclusion:**

This is the largest cohort‐level study in Spain to profile the analyses of biopsy samples of different tumors using NGS in routine clinical practice. Our findings showed that the use of NGS routinely provides good rates of informative results and can improve tumor characterization and identify a greater number of actionable mutations.

## Graphical abstract

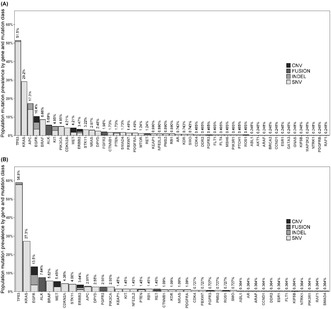

## 1 INTRODUCTION

The complexity of the cancer genome has been reported by numerous genomic researches over the last few years.^1^ In fact, the revolution in sequencing technology has improved the understanding of genome and the genetics.^2^

Next‐generation sequencing (NGS) is a high complex molecular technique that makes it possible to simultaneously test for a very large panel of genes by allowing parallel sequencing of numerous small nucleic acid fragments.^3^, ^4^ Compared with other sequencing modalities, NGS holds many advantages, for instance, high throughput to fully sequence all types of mutations for many genes (hundreds to thousands), the sensitivity and the speed.^1^, ^2^, ^5^ Moreover, testing in a single assay is both timely and cost‐effective as it can detect all four main classes of genomic alterations: base substitutions insert and deletions, copy number alterations, and rearrangements or fusions.^1^, ^2^, ^6^, ^7^

NGS has identified novel genetic alterations contributing to oncogenesis, cancer progression, and metastasis.^8^ These technologies are playing an increasingly important role in the study of different cancers such as prostate, pancreatic, small cell lung cancer, breast, ovarian, bladder cancer, renal cell carcinoma, or hematological neoplasms.^1^, ^2^, ^9^, ^10^ Thus, NGS has provided new insights into the search for cancer‐related genomic alterations in tumor cells and the understanding of tumor biology.^10^ The European Society for Medical Oncology (ESMO) recommends routine use of NGS for diverse cancers such as advanced non‐squamous non‐small‐cell lung cancer (NSCLC), prostate cancers, ovarian cancers, and cholangiocarcinoma.^9^ Also, the Asia‐Pacific Oncology Drug Development Consortium (APODDC) recommends NGS for the clinical use in metastatic NSCLC.^11^

Apart from identifying genetic and novel somatic mutations, another use of NGS is to improve rationally designed advanced personalized treatment of cancer.^2^ To date, the benefits of NGS are well known and many studies have applied NGS for personalized treatment of cancer.^1^

As part of precision oncology, NGS is a technology that can allow integration of molecular tumor profiles into clinical decision‐making and have increasingly replaced conventional techniques.^12^, ^13^ However, an analysis of methods in the Central Prospective Registry of Lung Cancer Biomarkers (LungPath) of the Spanish Society of Anatomic Pathology (SEAP) showed that the level of implementation of NGS was still low and mainly single‐gene conventional tests.^14^, ^15^ In an approach recently suggested by ESMO, it will increasingly become necessary to deploy multigene testing with NGS‐based techniques for the genomic profiling of cancer.^9^, ^16^ However, such support does not necessarily translate to equitable access to NGS within and across countries due to gaps in its implementation: varying practices in its use and access, which demonstrates different degrees of efficiency and deficiency across each European country.^12^

Although NGS has led to important findings in biomedical research and has already been implemented in clinical diagnostics, this technology has among its main obstacles to implementation the financial constraints, a lack of NGS testing capabilities, and the failure to include NGS testing in the guidelines.^8^, ^17^ Also, NGS accurate interpretation requires a multidisciplinary team with expertise in oncology, genetics, pathology, bioinformatics, and data storage.^1^, ^8^

The aim of this study is to describe the use of NGS to detect molecular alterations in Formalin‐Fixed Paraffin‐Embedded (FFPE) solid tumors from five Spanish hospitals. Specifically, the performance of NGS to detect molecular alterations in all tumors and lung cancers was assessed, and the frequency of actionable mutations detected in lung using NGS was evaluated.

## 2 MATERIALS AND METHODS

### 2.1 Patient selection and FFPE tissue collection (inclusion & exclusion criteria)

This is a cross‐sectional study performed at the Fundación Jiménez Díaz University Hospital from May 2021 and March 2022 during a routine molecular diagnostic throughput sequencing testing, including data from 537 tissue material samples corresponding to 493 consecutive routine clinical patients with a wide range of solid tumors (lung, colorectal, melanoma, gastrointestinal stromal, among others). NSCLC cases were selected independently of any histological subtypes or stage, with no limitation of smoking status, treatment line, organ function or ECOG performance status. All other tumor types, NGS was performed in advanced/metastatic stage cases. Patient samples were obtained from five Spanish centers, the Fundación Jiménez Díaz University Hospital (a 720 beds‐center with 358 new NSCLC cases per year), three hospitals from the National Health System in the Region of Madrid institutions (Villalba General University Hospital [217 beds and 79 NSCLC cases per year], Rey Juan Carlos University Hospital [350 beds and 155 NSCLC cases per year], Infanta Elena University Hospital [198 beds and 71 NSCLC cases per year]), and four small private hospitals clustered together. All patients gave written informed consent. All investigations followed standard operating procedures with the approval of the Fundación Jiménez Díaz University Hospital Ethics, Scientific Committee and Biobank Fundación Jiménez Díaz (PT20/00141) and were conducted in accordance with the ethical principles of the Declaration of Helsinki.

### 2.2 Nucleic acid isolation

FFPE tissues were sectioned 3‐μm thick for hematoxylin and eosin staining (Dako coverStainer, Agilent, Santa Clara, CA, USA) to ensure appropriate tumor‐cell content, greater than 30% of tumor cells. We used consecutive sections to extract both genomic DNA with AVENIO Tumor DNA Isolation Kit according to the manufacturer's instructions.

### 2.3 High‐throughput sequencing

DNA quantity was assessed using Qubit dsDNA HS Assay Kit according to the manufacturer's instructions. Quality of DNA samples was determined with AVENIO Tumor QC kit, samples with *Q*‐ratio value <0.04 were not eligible for assay performance. Sequencing libraries were prepared from 20 ng DNA, using AVENIO Tumor Tissue Targeted Kit (08456372001‐08456348001, Roche Diagnostics, Basel, Switzerland) contains 17 genes in the US National Comprehensive Cancer Network (NCCN) and other guidelines, and AVENIO Tumor Tissue Expanded Kit (08456321001‐08456356001, Roche Diagnostics, Basel, Switzerland) contains 17 genes plus 60 emerging biomarkers investigated in clinical trials The panels covers hotspot areas of genes with high relevance in solid tumors, allowing the identification of point mutations, insertions, deletions, copy number alterations, and gene translocations. Eight or 16 purified libraries per run were pooled and sequenced on an Illumina NextSeq 500 (Illumina, San Diego, CA, USA), using the 300‐cycle NextSeq 500/550 Mid Output v2 kit or the 300‐cycle NextSeq High Output kit, respectively, in paired‐end mode (2 × 151 cycles). Data analysis for variant calling and annotation were performed using the AVENIO Oncology Analysis Software (Roche Diagnostics), a minimum sequencing depth of 1000× was considered appropriate, and variants with an allelic frequency (AF) less than 5.00% were filtered for single nucleotide variant (SNV), intergenic and intragenic fusions, and >5 copies for copy number variation (CNVs).

### 2.4 Immunohistochemistry

Serial FFPE tissue sections of 3‐μm thick were pretreated and stained with PD‐L1 IHC 22C3 PharmDx (Agilent/Dako, Santa Clara, CA, USA) according to the manufacturer's instructions, in order to determine the protein expression of PD‐L1 in patients with NSCLC for immunotherapy decision. Two different pathologists independently assessed the presence or absence of tumor‐cell immunoreactivity and positive controls. Evaluation of PD‐L1 was done by calculation of the Tumor Proportion Score (TPS).

### 2.5 Main outcomes

We used the informative results rate as a measure of sequencing performance. Mutated or non‐mutated results were considered informative. Non‐informative results were those obtained from tissue samples deemed not evaluable for different reasons: insufficient tumor representation in the sample (<30% tumor cell or <2 mm^2^ of tumor area), low genomic DNA sample quality (*Q*‐ratio < 0.04) or sequencing not measurable, and samples with inadequate QC metrics With these data, we were able to determine the performance of AVENIO for the detection of molecular alterations in FFPE solid tumors. Then, with informative results, samples with mutated o non‐mutated NGS results, we described the most frequently altered genes and mutations in FFPE solid tumors.

We also deeply analyzed the findings of actionable mutations in samples of lung cancer for considering treatment decisions. The variant classification and level of actionability were assessed based on the Association for Molecular Pathology and College of American Pathologists (AMP and CAP) standardization guidelines^18^ and the European Society for Medical Oncology (ESMO) Scale for Clinical Actionability of molecular Targets (ESCAT) guideline.^9^ Therefore, the first group comprising ESCAT level I mutations with targeted therapies currently reimbursed in Spain was analyzed. A second group also comprises actionable mutations defined as ESCAT I with non‐reimbursed targeted therapies in Spain and ESCAT level II, in addition to the previous group.^9^, ^19^

### 2.6 Statistical analysis

Medians and quartiles or absolute and relative frequencies were used to describe quantitative and categorical variables, respectively. No imputation method was used to supply missing data. The number of missing cases is always indicated. The exact binomial test was used to obtain 95% confidence intervals (CI) for proportions of prevalence of single mutations, informative results, and actionable mutations.

Univariable and multivariable logistic analysis was conducted to assess the impact of sample characteristics on informative results rate for all tumors and for lung tumors, and on actionable mutations rate found in lung tumor samples. Results are presented by means of odds ratio and 95% CI.

The R programming language^20^ and the RStudio environment^21^ were used for the data analysis. All tests were two‐sided at a significant level of *α* = 0.05.

## 3 RESULTS

### 3.1 Clinicopathological characteristics of patients and samples

The characteristics of the patients are summarized in Table 1.

**TABLE 1:** Characteristics of patients with tissue material samples available.

Characteristics	All tumors *N* = 493	Lung tumors *N* = 337
Female	178 (36.1)	113 (33.5)
Age, years	68 (61, 74)	68 (62, 75)
Smoking habits		
Current smoker	‐	131 (40.2)
Former smoker	‐	161 (49.4)
Non‐smoker	‐	34 (10.4)

*Note*: Categorical variables are described with frequencies and percentages. For age, median and percentiles of 25% and 75% are shown. Missing values: smoking habits (*n* = 11).

Since there may be several samples from the same patient, Table 2 shows the characteristics of the 537 all tumor samples and of the 365 samples of lung tumors (detailed in Table S1). The most common type of tumor was non‐small cell lung carcinoma (68.4% of all tumors), and with a lower frequency colorectal carcinoma, melanoma, and gastrointestinal stromal tumor (GIST). The most common sample collection procedure was diagnostic biopsy (58.8% for all tumors), and waiting time until receiving NGS results (P25, P75) was 13 (9, 15) natural days, for all tumor samples.

**TABLE 2:** Characteristics of the analyzed samples.

Characteristics	All tumors *N* = 537	Lung tumors *N* = 365
Center		
1	284 (52.9)	204 (55.9)
2	62 (11.5)	36 (9.86)
3	123 (22.9)	74 (20.3)
4	60 (11.2)	44 (12.1)
5	8 (1.49)	7 (1.92)
Sample collection procedure		
Diagnostic biopsy	307 (58.8)	219 (61.9)
Surgical specimen	157 (30.1)	82 (23.2)
Cytology	58 (11.1)	53 (15.0)
Tumor type		
Non‐small cell lung carcinoma (NSCLC)	365 (68.4)	365 (100)
Colorectal carcinoma	85 (15.9)	‐
Melanoma	32 (5.99)	‐
Gastrointestinal stromal tumor (GIST)	39 (7.30)	‐
Others^a^	13 (2.43)	‐
Lung tumor type		
Adenocarcinoma, large cell or undifferentiated	‐	251 (69.5)
Squamous cell carcinoma	‐	106 (29.4)
Others	‐	4 (1.11)
Informative results		
Informative	404 (75.2)	275 (75.3)
Non‐informative	133 (24.8)	90 (24.7)
Mutational status		
Mutation found	351 (65.4)	239 (65.5)
No mutation found	53 (9.87)	36 (9.86)
Not evaluable	133 (24.8)	90 (24.7)
PD‐L1 22C3 expression level (TPS), %	‐	‐
Negative [0, 1)	‐	130 (37.2)
Positive non‐overexpressed [1, 50)	‐	99 (28.4)
Positive overexpressed ≥ 50	‐	120 (34.4)

*Note*: Categorical variables are described with frequencies and percentages. Missing values: sample collection procedure (*n* = 15 in all tumors, *n* = 11 in lung tumors); tumor type (*n* = 3); lung tumor type (*n* = 4); PD‐L1 levels TPS (*n* = 16).

^a^ Others include: Annus, esophagus, larynx, salivary gland, breast, ovary, pancreas, prostate, biliary tract, and unknown origin.

Among lung cancers, adenocarcinoma, large cell, or undifferentiated were the most common subtype (69.5% of lung tumors), diagnostic biopsy again also the most common sample collection procedure (61.9% for lung tumors).

For all tumor samples, NGS analysis provided 75.2% informative results, of which 86.9% presented, at least, one gene mutation (representing 65.4% of all samples analyzed). Therefore, 24.8% of the samples were not evaluable, either due to insufficient sample (21.1% of the non‐informative samples; 5.21% of the samples analyzed) or due to low DNA sample quality for AVENIO NGS (75.2% of the non‐informative samples; 18.6% of samples tested) or by non‐evaluable sequencing (3.80% of non‐informative samples; 0.93% of all samples analyzed).

In lung tumor samples, informative results were very similar to that of all tumor samples, as shown in Table 2. The causes of the 24.7% non‐evaluable samples were insufficient sample (24.4% of the non‐informative samples; 6.03% of lung tumor samples), low sample quality (72.2% of the non‐informative samples; 17.8% of samples tested), or non‐evaluable sequencing (3.33% of non‐informative samples; 0.82% of lung tumor samples).

### 3.2 Informative results rate (AVENIO performance)

Using the percentage of informative results as a measure of the performance of NGS, a performance rate (95% CI) of 75.2% (71.4%, 78.8%) and 75.3% (70.6%, 79.7%) was obtained for all tumor samples and lung cancer samples, respectively.

Descriptive results of performance rate by characteristics of tumor samples and univariable OR are detailed in Tables S2 and S3.

Multivariable logistic regression analysis of performance rate in all tumor samples showed that the NGS achievement was higher for the samples from center 1 and significantly lower for those from centers 3 and 4. Sample collection procedure was also associated with significant differences in performance, being surgery the procedure most likely to provide informative results. Cytology and diagnostic biopsy also showed good rates of informative results, but no statistically significant differences were observed. Finally, GISTs were the tumors whose samples had a significantly lower performance than lung carcinomas (Table 3).

**TABLE 3:** Multivariable analysis of informative results rate.

Characteristic	All tumors		Lung tumors	
	OR (95% CI)	*p*‐value	OR (95% CI)	*p*‐value
Center (vs. 1)				
2	0.55 (0.27, 1.11)	0.093	0.72 (0.26, 2.00)	0.534
3	0.16 (0.10, 0.26)	<0.001	0.13 (0.07, 0.26)	<0.001
4	0.46 (0.23, 0.91)	0.026	0.46 (0.20, 1.09)	0.077
5	n.e.	‐	n.e.	‐
Sample collection procedure (vs. diagnostic biopsy)				
Surgery	2.85 (1.61, 5.07)	<0.001	‐	‐
Cytology	1.03 (0.52, 2.06)	0.932	‐	‐
Sample collection procedure (vs. CNB or EBUS‐CNB)				
Other biopsies	‐	‐	1.05 (0.51, 2.18)	0.890
Surgery	‐	‐	4.02 (1.53, 10.6)	0.005
Cytology	‐	‐	1.05 (0.45, 2.46)	0.909
Tumor type (vs. lung carcinoma)				
Colorectal carcinoma	1.39 (0.72, 2.69)	0.321	‐	‐
Melanoma	1.05 (0.42, 2.63)	0.920	‐	‐
Gastrointestinal stromal tumor	0.36 (0.16, 0.78)	0.010	‐	‐
Others	0.66 (0.18, 2.40)	0.529	‐	‐
Tumor type (vs. adenocarcinoma, large cell or undifferentiated)				
Squamous cell carcinoma	‐	‐	1.16 (0.62, 2.16)	0.648
Smoking habits (vs. current smoker)				
Former smoker	‐	‐	1.41 (0.78, 2.54)	0.259
Non‐smoker	‐	‐	1.98 (0.70, 5.57)	0.197
Stage (vs. IA)				
IB	‐	‐	1.34 (0.42, 4.24)	0.621
IIA	‐	‐	0.89 (0.14, 5.43)	0.897
IIB	‐	‐	1.83 (0.43, 7.75)	0.412
IIIA	‐	‐	1.29 (0.36, 4.64)	0.699
IIIB‐C	‐	‐	3.31 (0.97, 11.2)	0.056
IV	‐	‐	1.57 (0.60, 4.15)	0.360

Abbreviations: CI, confidence interval; CNB, core needle biopsy; EBUS, endobronchial ultrasound; n.e, not estimable; OR, odds‐ratio; Ref., reference level.

Regarding lung cancer patients, similar results were obtained. Significantly lower performance was observed for samples from center 3 compared with center 1. Surgery was the collection procedure most likely to provide informative results, and no differences were observed between samples obtained by CNB, cytology, and other biopsies. Finally, no differences in informative rate were also observed by type of tumor, smoking habit, or stage of cancer (Table 3).

### 3.3 Metrics results

Metric values were available for 404 samples of any tumor and for 275 samples of lung tumor.

Median (P25, P75) of DNA mass was 27.9 (21.4, 42.9) ng for all tumor samples and 25.6 (20.8, 39.0) ng for lung samples. *Q*‐ratio was 0.55 (0.30, 0.86) and 0.63 (0.34, 0.91) for all tumor and lung tumor samples, respectively.

The 5th percentile of unique depth was over 500 in 73% of all tumor samples and in 74.2% of lung tumor samples. See more metrics results in Table S4.

### 3.4 Detection of genomic alterations

Excluding benign or likely benign mutations, 727 pathogenic and likely pathogenic, or variant of unknown significance (VUS) mutations were found in all tumor samples, of which 19% were VUS. In NSCLC, 448 pathogenic, likely pathogenic, or VUS mutations were found, of which 16.3% were VUS (Table 4). Mostly, mutational class was SNV both for all tumor samples (85.3%) and for lung tumor samples (81.9%).

**TABLE 4:** Mutational findings.

Characteristics	All tumors *N* = 727	Lung tumors *N* = 448
Pathogenicity		
Pathogenic and likely pathogenic	589 (81.0)	375 (83.7)
VUS	138 (19.0)	73 (16.3)
Mutational class		
CNV (Copy number variation)	28 (3.86)	24 (5.36)
Gene fusion	34 (4.68)	34 (7.59)
INDEL (Insertion–deletion)	45 (6.20)	23 (5.13)
SNV (single nucleotide variant)	619 (85.3)	367 (81.9)

*Note*: Categorical variables are described with frequencies and percentages. Missing values: mutational class (*n* = 1 in all tumors).

Abbreviation: VUS, variant of unknown significance.

**FIGURE 1:**
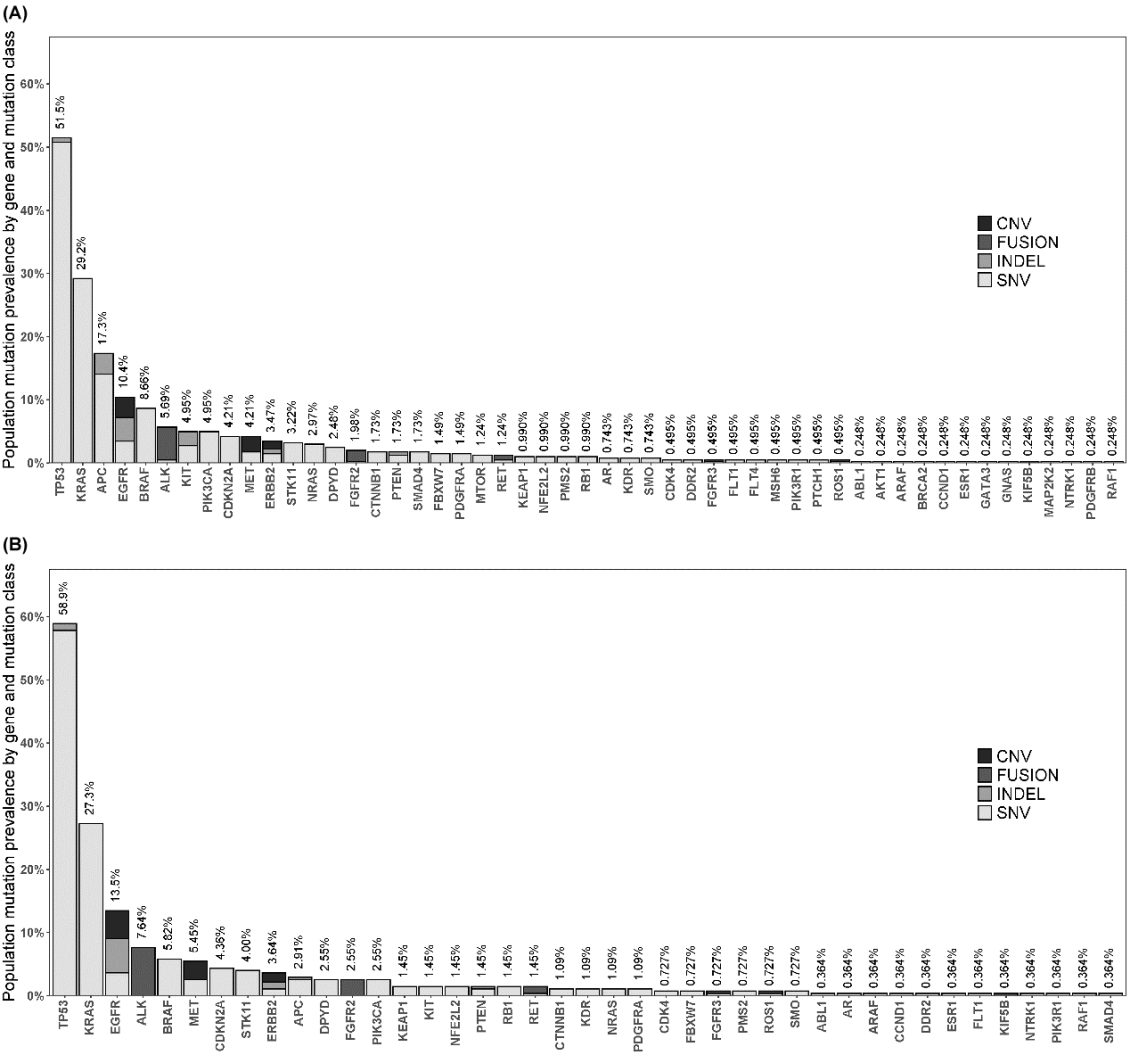
Population prevalence of genomic alterations by histological subtype. (A) All tumor samples. (B) Lung tumor samples. CNV, copy number variation; INDEL, insertion–deletion; SNV, single nucleotide variant.

Over the number of evaluable samples from any tumor, the mutated genes with the highest prevalence (95% CI) were as follows: TP53, 51.5% (46.5%, 56.5%); KRAS, 29.2% (24.8%, 33.9%); APC, 17.3% (13.8%, 21.4%); EGFR, 10.4% (7.6%, 13.8%); and BRAF, 8.70% (6.10%, 11.8%). In lung tumor evaluable samples were: TP53, 58.9% (52.8%, 64.8%); KRAS, 27.3% (22.1%, 32.9%); EGFR, 13.5% (9.70%, 18.1%); and ALK, 7.60% (4.80%, 11.4%) (Figure 1; Tables S5 and S6).

Among INDEL and SNV mutations, the most common principal variant was missense variant both for all tumor samples (69.8%) and lung tumor samples (71.8%). See all principal variants in Table S7.

See Table S8 for descriptive results of sequencing depth measures and number of exons in principal variants by mutational class.

### 3.5 Analysis of actionable mutations in lung tumor samples

The prevalence (95% CI) of actionable mutations among the first group analyzed (ESCAT I mutations with targeted therapies currently reimbursed in Spain) was 13.5% (9.70%, 18.1%). Including those that are ESCAT I with non‐reimbursed targeted therapies in Spain and ESCAT II, the prevalence was 26.5% (21.4%, 32.2%). Individually, the most common actionable mutations were mutations in the KRAS gene hotspots p.Gly12Cys (8.36%) and in the EGFR gene (8.00%) (Table 5).

**TABLE 5:** Descriptive results of actionable mutations in lung cancer by ESCAT level and reimbursement status in Spain.

	Actionable mutation	*n* (%)
ESCAT I (with reimbursed targeted therapies in Spain)	EGFR^a^	22 (8.00)
	ALK (Gene Fusion)	10 (3.64)
	ROS1 (Gene Fusion)	1 (0.36)
	RET (Gene Fusion)	2 (0.73)
	MET (Exon 14 skipping mutation*)*	2 (0.73)
ESCAT I (with not reimbursed targeted therapies in Spain) and ESCAT II	BRAF^V600E^	4 (1.45)
	KRAS^G12C^	23 (8.36)
	RET (SNV)	1 (0.36)
	MET (Amplifications)	8 (2.91)
	ERBB2 (Hotspot mutations and amplifications)	4 (1.45)
	NTRK1 (Gene Fusion)	0 (0.00)
ESCAT I with reimbursed targeted therapies in Spain		37 (13.5)
Actionable mutation ESCAT I‐II		73 (26.5)

Abbreviation: ESCAT, ESMO Scale for Clinical Actionability of molecular Targets.

^a^ Common mutations (Del19, L858R) and Acquired T790M exon 20 mutation; Uncommon EGFR mutations (G719X in exon 18, L861Q in exon 21, S768I in exon 20) Exon 20 insertions.

Univariable OR for actionable mutations are detailed in Tables S9 and S10.

Multivariable logistic regression analysis showed that it is more likely to find actionable mutations in lung tumor samples from non‐smokers and patients with adenocarcinoma, large cell, or undifferentiated tumor, both for actionable mutations with specific targeted therapy available (Table 6), and when those with novel treatments under development were also included (Table 7). No differences were observed for the rest of characteristics.

**TABLE 6:** Multivariable analysis lung cancer ESCAT I actionable mutations with reimbursed targeted therapy in Spain.

Characteristic	OR (95% CI)	*p*‐value
Center (vs. 1)		
2–3	0.39 (0.10; 1.61)	0.194
4	0.39 (0.09; 1.72)	0.213
5	n.e.	‐
Sample collection procedure (vs. CNB or EBUS‐CNB)		
Other diagnostic biopsies	2.48 (0.70; 8.74)	0.159
Surgical specimen	2.00 (0.61; 6.64)	0.255
Cytology	1.04 (0.23; 4.72)	0.959
Tumor type (vs. adenocarcinoma, large cell, or undifferentiated)		
Squamous cell carcinoma	0.08 (0.01; 0.66)	0.018
Smoking habits (vs. current/former smoker)		
Non‐smoker	11.4 (4.22; 30.5)	<0.001
Stage (vs. IA)		
IB	1.84 (0.35; 9.58)	0.470
IIA–B	0.27 (0.02; 3.51)	0.315
IIIA	0.44 (0.03; 6.25)	0.542
IIIB–C	1.44 (0.21; 9.71)	0.706
IV	1.24 (0.25; 6.18)	0.789
PD‐L1 expression (vs. negative)		
Positive non‐overexpressed [1, 50)	2.44 (0.83; 7.15)	0.105
Positive overexpressed ≥50	1.34 (0.44; 4.06)	0.608

Abbreviations: CI, confidence interval; CNB, core needle biopsy; EBUS, endobronchial ultrasound; n.e, not estimable; OR, odds‐ratio; Ref., reference level.

**TABLE 7:** Multivariable analysis of lung cancer actionable mutations ESCAT I‐II.

Characteristic	OR (95% CI)	*p*‐value
Center (vs. 1)		
2	0.83 (0.27; 2.60)	0.750
3	0.48 (0.16; 1.46)	0.194
4	0.86 (0.32; 2.31)	0.757
5	n.e.	‐
Sample collection procedure (vs. CNB or EBUS‐CNB)		
Other diagnostic biopsies	1.23 (0.52; 2.92)	0.634
Surgical specimen	0.96 (0.40; 2.27)	0.924
Cytology	1.51 (0.57; 4.03)	0.408
Tumor type (vs. adenocarcinoma, large cell or undifferentiated)		
Squamous cell carcinoma	0.20 (0.08; 0.51)	<0.001
Smoking habits (vs. current/former smoker)		
Non‐smoker	4.62 (1.99; 10.7)	<0.001
Stage (vs. IA)		
IB	0.94 (0.26; 3.38)	0.919
IIA	3.86 (0.51; 29.4)	0.192
IIB	1.35 (0.26; 7.11)	0.721
IIIA	0.73 (0.13; 4.08)	0.721
IIIB–C	1.34 (0.34; 5.34)	0.675
IV	1.46 (0.46; 4.67)	0.524
PD‐L1 expression (vs. negative)		
Positive non‐overexpressed [1, 50)	1.64 (0.75; 3.57)	0.215
Positive overexpressed ≥50	1.37 (0.64; 2.92)	0.416

Abbreviations: CI, confidence interval; CNB, core needle biopsy; EBUS, endobronchial ultrasound; n.e, not estimable; OR, odds‐ratio; Ref., reference level.

## 4 DISCUSSION

We analyzed the performance of NGS and the frequency of actionable mutations in a large sample of FFPE solid tumors, which provides useful and necessary results to characterize the tumor and make the best therapeutic decision available, identifying all types of molecular alterations, especially those with specific targeted therapies available.

The results obtained in our study show that NGS analysis provided 75.2% informative results, of which 86.9% had some type of mutation (for all tumor samples). Performance rate in lung cancer samples was almost the same as in the overall tumor samples. Low‐quality samples are mainly from non‐formation samples. Also, we found that surgical samples were most likely to provide informative results. Regarding the rate of actionable mutations in lung cancer, we found that using NGS could contribute to optimize the detection of these mutations, observing a prevalence of actionable mutations with a specific targeted therapy available of 12.0%, being KRAS p.Gly12Cys the most frequent actionable mutation. Also, we found that it is more likely to find actionable mutations in samples from non‐smokers.

To our knowledge, this is the largest cohort‐level study in Spain to profile the analyses of cancer biopsy samples using NGS in routine clinical practice. The recent study published by Simarro et al.^22^ evaluated the implementation of NGS for lung cancer biopsy samples in routine clinical practice,^22^ in contrast to our study which analyzed biopsy samples of another cancer in addition to lung cancer, using NGS routinely.

Provencio et al.^23^ in an observational, prospective, registry‐based study of The Thoracic Tumours Registry (TTR), that included 9239 Spanish patients diagnosed with lung cancer and other thoracic tumors (2016–2020), considered that national strategies are urgently needed to implement NGS in an integrated and cost‐effective way in lung cancer and analyzed the global testing of EGFR (67.0%), ALK (54.9%), and ROS1 (30.3%).^23^

There are also other observational studies with some parallels to ours, considering the difference in the number and characteristics of the patients. The aforementioned study by Simarro et al.^22^ reported the integration of NGS studies (350 patients) into a Spanish reference public healthcare hospital, founding that TP53 (51.0%), KRAS (26.6%), and EGFR (12.9%) were the most frequently mutated genes. Moreover, they found that actionable genetic alterations were significantly more frequent in never‐smokers (87.7%, *p* < 0.001),^22^ as we have found in our study. Also, in study of Sorin et al.^24^ it was described the genomic landscape of patients with lung cancer (*n* = 997) with NGS in a Canadian hospital, founding a higher prevalence of KRAS mutations (39.2%) compared with most geographical locations, which proved the important to assess institutional rates of actionable driver mutations to help guide governing bodies.^24^ On the other hand, Garcia‐Casado et al.^25^ and Sargas et al.^26^ are prospective studies of epithelial ovarian cancer and acute myeloid leukemia, respectively, that provide an extensive molecular characterization, risk stratification, ensuring technical quality and equity in access to NGS studies.^25^, ^26^ In non‐European countries, the decision analytic model of Matsuda et al.^27^ that was used to estimate the budgetary impact of SSG testing versus NGS in newly diagnosed patients with advanced NSCLC in Japan, showing that the adoption of NGS instead of SSG would shorten the average turnaround time of testing (14.32 vs. 18.10, respectively).^27^

In order to analyze the current biomarker testing practices in solid tumors and identify country‐specific shortcomings, the International Quality Network for Pathology (IQN Path), the European Cancer Patient Coalition (ECPC), and the European Federation of Pharmaceutical Industries and Associations (EFPIA), together with a consortium of industry and academic partners, have conducted research across the 27 European countries (EU27) and the United Kingdom (UK). They found limits to the access to single biomarker tests were identified in many European countries. For instance, in Southern and Eastern Europe due to lower levels of public reimbursement for testing and variability in order rates. On the other hand, multi‐biomarker test access is highly varied ranging from 0.00% in Slovakia to more than 50.0% in Denmark and the Netherlands. In Spain (2.00% of uptake), the use of NGS panels is limited by the availability of funding and more than 25.0% of the cost of NGS testing must be covered either by the patient or by pharmaceutical sponsors. Even though, Spanish patients rated their satisfaction with the information received as high.^17^

This argument is supported by the study by Horgan et al.^12^ which claims that many European patients with cancer are not fully benefiting from NGS‐driven approaches due to gaps in its implementation. In addition, specialist advisory bodies, known as molecular tumor boards (MTBs), have different constitutions and aims from country to country. In Spain, MTBs are mainly found at regional level within large hospitals that use NGS. On the other hand, European NSCLC guidelines recommend NGS‐based genomic testing for EGFR, ALK, ROS1, BRAF NTRK, and KRAS, while in Spain, guidelines recommend molecular testing of EGFR, ALK, ROS1, and BRAF, but only suggest additional testing of genes such as KRAS and NTRK if previous biomarker testing yields negative results, we reflect in our study.^12^ In colorectal cancer, ESMO suggests that an optimal gene panel should detect KRAS, NRAS, BRAF, NTRK, and ERBB2 amplification, although NGS is still only considered for research purposes.^9^

There is great potential for the application of NGS in disease management and treatment, genetic counseling, and risk assessment. NGS can also be used for molecular diagnosis of genetic and infectious diseases, carrier detection, medical genetics and pharmacogenomics, molecular diagnosis of cancer, and prognostics.^2^

Although NGS has led to important findings in biomedical research and has already been implemented in clinical diagnostics, this technology has among its main obstacles to implementation the financial constraints, a lack of NGS testing capabilities, and the failure to include NGS testing in the guidelines.^8^, ^17^ Also, with NGS technology will also have to be considered to have the full picture of the disease, for example, it requires a multidisciplinary team with expertise in oncology, genetics, pathology, bioinformatics, and data storage.^1^, ^8^

For that reason and with a future perspective, national strategies must aim to regulate NGS standardization and quality, ensure the development in Europe of precision medicine, and educate specialist medical oncology in the use of NGS.^12^, ^13^, ^17^ The above reinforces the need to perform cost‐effectiveness analyses on the use of NGS. Furthermore, frameworks differ across Europe, with some countries placing more of an emphasis on clinical outcomes, and others on cost‐effectiveness.^12^ In recent years, the need to perform cost‐effectiveness analyses on the use of NGS has been reinforced with the publication of literature reviews which concluded that NGS was cost‐effective.^28^, ^29^ In addition, some cost‐effectiveness analyses have been carried out in our country.^30^, ^31^ In fact, a recent Spanish cost‐effectiveness analysis shows that the use of NGS over SgT in Spanish reference centers for the molecular diagnosis of patients with metastatic NSCLC would be a cost‐effective strategy.^30^

Our study has some limitations. The compilation of samples is lacking from further tumor types and probably results extrapolation should be done with caution. Although the application of the comprehensive high‐throughput panel permitted a confident molecular characterization, some biomarkers may are not present in the used panel. Nevertheless, the kit used to characterize the molecular profiling of the study samples is one of the tests that detect the widest range of alterations among commercially available tests. Also, at the time of analysis, EGFR, ALK, ROS1, RET, and MET (exon 14 skipping); were the only actionable mutations with reimbursed targeted therapies; however, this will be changing in the coming years. However, as far as we know, this is the largest study providing real‐world evidence of the impact of sample characteristics on the informative results rate in both all tumor samples and lung tumor samples, and on the probability of finding actionable mutations in lung tumor samples. Furthermore, in relation to the description of the actionable mutations detected, it should be considered that the selection of genes was carried out on the basis of targeted therapies currently reimbursed in Spain.

In summary, NGS's implementation has been a milestone for application of precision medicine by opening to a wider range of patients, and our study provides valuable information from the routine use of NGS on FFPE samples from a large cohort of patients with various tumor types. In any case, further studies are needed to assess the cost‐effectiveness of NGS versus single‐gene testing for its routine adoption in hospital pathology laboratories.

## AUTHOR CONTRIBUTIONS

**Sandra Zazo:** Conceptualization (equal); data curation (lead); methodology (equal); supervision (equal); validation (equal); writing – review and editing (equal). **Sandra Pérez‐Buira:** Conceptualization (equal); data curation (equal); methodology (equal); supervision (equal); validation (equal); writing – review and editing (equal). **Nerea Carvajal:** Conceptualization (equal); data curation (supporting); methodology (supporting); supervision (equal); validation (equal); writing – review and editing (equal). **Jenifer Plaza‐Sánchez:** Validation (equal); writing – review and editing (equal). **Rebeca Manso:** Validation (equal); writing – review and editing (equal). **Nuria Pérez‐González:** Validation (equal); writing – review and editing (equal). **Carolina Dominguez:** Validation (equal); writing – review and editing (equal). **Iván Prieto‐Potin:** Validation (equal); writing – review and editing (equal). **Jaime Rubio:** Validation (equal); writing – review and editing (equal). **Manuel Dómine:** Validation (equal); writing – review and editing (equal). **Virginia Lozano:** Conceptualization (equal); data curation (supporting); formal analysis (supporting); funding acquisition (lead); project administration (equal); supervision (supporting); validation (equal); writing – review and editing (equal). **Patricia Mohedano:** Conceptualization (equal); data curation (supporting); formal analysis (supporting); funding acquisition (supporting); project administration (equal); supervision (supporting); validation (equal); writing – review and editing (equal). **David Carcedo:** Conceptualization (equal); data curation (equal); formal analysis (lead); methodology (equal); writing – original draft (lead); writing – review and editing (equal). **Rafael Carias:** Validation (equal); writing – review and editing (equal). **Federico Rojo:** Conceptualization (lead); data curation (equal); formal analysis (equal); methodology (equal); supervision (lead); validation (lead); writing – review and editing (lead).

## FUNDING INFORMATION

This study was funded by Roche Farma S.A. Roche Farma S.A played no role in the design of the study and collection, analysis, and interpretation of data and in writing the manuscript. This research was also partially funded by the Spanish Ministry of Economy and Competitiveness (MINECO, AES program), grant number PI21/00142, and Fundación Jiménez Díaz Biobank PT20/00141.

## CONFLICT OF INTEREST STATEMENT

Jaime Rubio declares to have received personal funds for consultation and advisor role by Roche, AstraZeneca, MSD Oncology, and BMS. Manuel Domine declares to have received personal funds for consultation and advisor role by Roche, AstraZeneca, MSD Oncology, BMS, Lilly, Pfizer and Abbvie; and received travel funds by AstraZeneca, Pfizer, and BMS. Federico Rojo declares to have received personal funds for advisor role by Roche, AstraZeneca, Daiichi Sankyo, MSD, BMS, Pfizer, GSK, Novartis, Amgen, Merck, and Sophia Genetics; and received travel funds by Roche and BMS. Sandra Zazo, Sandra Pérez, Nerea Carvajal, Jenifer Plaza‐Sánchez, Rebeca Manso, Nuria Pérez‐González, Carolina Dominguez, Iván Prieto‐Potin, and Rafael Carias have no conflicts of interest to declare. David Carcedo is an employee of Hygeia Consulting which received funding from Roche to conduct the analysis. Virginia Lozano and Patricia Mohedano are employees of Roche.

## ETHICS STATEMENT

The study was conducted in accordance with the Declaration of Helsinki and approved by the Fundación Jiménez Díaz University Hospital Ethics and Scientific Committee (protocol code PIC209‐21).

## Supporting information


Data S1.

## Data Availability

Datasets used in the current study may be available to qualified researchers on a reasonable request.
