# Supplementary material for: Actionable mutational profiling in solid tumors using hybrid‐capture‐based next‐generation sequencing in a real‐world setting in Spain
Source: Cancer Med. 2024 Jan 11;13(3):e6827. doi: 10.1002/cam4.6827 (PMC10905216; doi:10.1002/cam4.6827)
Supplement: Supplementary file 1 — Data S1. [file CAM4-13-e6827-s001.docx]

# Supplementary material

**Table S1**. Characteristics of the analyzed lung cancer samples.

| **Characteristics** | **Lung tumors**  **N = 365** | **Adenocarcinoma, large cell or undifferentiated**  **N=251** | **Squamous cell carcinoma**  **N=106** | **Others**  **N=4** |  |
| --- | --- | --- | --- | --- | --- |
| Center |  |  |  |  |  |
| *1* | 204 (55.9) | 147 (58.6) | 55 (51.9) | 2 (50.0) |  |
| *2* | 36 (9.86) | 20 (7.97) | 14 (13.2) | 2 (50.0) |  |
| *3* | 74 (20.3) | 50 (19.9) | 24 (22.6) | 0 (0.00) |  |
| *4* | 44 (12.1) | 31 (12.4) | 13 (12.3) | 0 (0.00) |  |
| *5* | 7 (1.92) | 3 (1.20) | 0 (0.00) | 0 (0.00) |  |
| Sample collection procedure |  |  |  |  |  |
| *Diagnostic biopsy* | 219 (61.9) | 139 (57.0) | 77 (72.6) | 3 (75.0) |  |
| *Surgical specimen* | 82 (23.2) | 62 (25.4) | 19 (17.9) | 1 (25.0) |  |
| *Cytology* | 53 (15.0) | 43 (17.6) | 10 (9.43) | 0 (0.00) |  |
| Informative results |  |  |  |  |  |
| *Informative* | 275 (75.3) | 190 (75.7) | 80 (75.5) | 3 (75.0) |  |
| *Non-informative* | 90 (24.7) | 61 (24.3) | 26 (24.5) | 1 (25.0) |  |
| Mutational status |  |  |  |  |  |
| *Mutation found* | 239 (65.5) | 166 (66.1) | 68 (64.2) | 3 (75.0) |  |
| *No mutation found* | 36 (9.86) | 24 (9.56) | 12 (11.3) | 0 (0.00) |  |
| *Not evaluable* | 90 (24.7) | 61 (24.3) | 26 (24.5) | 1 (25.0) |  |
| PD-L1 22C3 expression level (TPS), % | - |  |  |  |  |
| *Negative [0, 1)* | 130 (37.2) | 92 (37.6) | 36 (36.0) | 2 (50.0) |  |
| *Positive non-overexpressed [1, 50)* | 99 (28.4) | 68 (27.8) | 30 (30.0) | 1 (25.0) |  |
| *Positive overexpressed ≥50* | 120 (34.4) | 85 (34.7) | 34 (34.0) | 1 (25.0) |  |
| *Categorical variables are described with frequencies and percentages. Missing values: lung tumor type (n=4); sample collection procedure (n=11 in all lung tumors, n=7 in adenocarcinoma, large cell or undifferentiated); PD-L1 levels TPS (n=16 in all lung tumors, n=6 in adenocarcinoma, large cell or undifferentiated, n=6 in squamous cell carcinoma).* | | | | | |

**Table S2**. Univariable analysis of informative results rate for all tumor samples

| **Characteristic** | **Non-informative**  **N = 133** | **Informative**  **N = 404** | **OR (95% CI)** | **p-value** |  |
| --- | --- | --- | --- | --- | --- |
| Center |  |  |  |  |  |
| *1* | 41 (14.4) | 243 (85.6) | Ref. | Ref. |  |
| *2* | 14 (22.6) | 48 (77.4) | 0.58 (0.30, 1.18) | 0.126 |  |
| *3* | 60 (48.8) | 63 (51.2) | 0.18 (0.11, 0.29) | <0.001 |  |
| *4* | 16 (26.7) | 44 (73.3) | 0.46 (0.24, 0.92) | 0.028 |  |
| *5* | 2 (25.0) | 6 (75.0) | 0.48 (0.10, 3.76) | 0.432 |  |
| Sample collection procedure |  |  |  |  |  |
| *Diagnostic biopsy* | 89 (29.0) | 218 (71.0) | Ref. | Ref. |  |
| *Surgical specimen* | 23 (14.6) | 134 (85.4) | 2.36 (1.44, 4.00) | <0.001 |  |
| *Cytology* | 18 (31.0) | 40 (69.0) | 0.90 (0.50, 1.70) | 0.747 |  |
| Tumor type |  |  |  |  |  |
| *Lung carcinoma* | 90 (24.7) | 275 (75.3) | Ref. | Ref. |  |
| *Colorectal carcinoma* | 16 (18.8) | 69 (81.2) | 1.40 (0.79, 2.62) | 0.256 |  |
| *Melanoma* | 8 (25.0) | 24 (75.0) | 0.97 (0.43, 2.40) | 0.944 |  |
| *Gastrointestinal stromal tumor* | 14 (35.9) | 25 (64.1) | 0.58 (0.29, 1.20) | 0.140 |  |
| *Others* | 5 (38.5) | 8 (61.5) | 0.52 (0.17, 1.80) | 0.284 |  |
| Categorical variables are described with frequencies and percentages. CI, confidence interval; OR, odds-ratio; Ref., reference level. Missing values: sample collection procedure (n=3 in non-informative, n=12 in informative); tumor type (n=3 in informative) | | | | | |

**Table S3**. Univariable analysis of informative results rate for lung tumor samples

| **Characteristic** | **Non-informative**  **N = 90** | **Informative**  **N = 275** | **OR (95% CI)** | **p-value** |  |
| --- | --- | --- | --- | --- | --- |
| Center |  |  |  |  |  |
| *1* | 28 (13.7) | 176 (86.3) | Ref. | Ref. |  |
| *2* | 7 (19.4) | 29 (80.6) | 0.65 (0.27, 1.77) | 0.379 |  |
| *3* | 41 (55.4) | 33 (44.6) | 0.13 (0.07, 0.24) | <0.001 |  |
| *4* | 12 (27.3) | 32 (72.7) | 0.42 (0.20, 0.95) | 0.038 |  |
| *5* | 2 (28.6) | 5 (71.4) | 0.38 (0.08, 3.10) | 0.322 |  |
| Sample collection procedure |  |  |  |  |  |
| *CNB or EBUS-CNB* | 45 (33.3) | 90 (66.7) | Ref. | Ref. |  |
| *Other diagnostic biopsies* | 18 (21.7) | 65 (78.3) | 1.79 (0.96, 3.45) | 0.066 |  |
| *Surgical specimen* | 8 (9.76) | 74 (90.2) | 4.53 (2.10, 11.0) | <0.001 |  |
| *Cytology* | 16 (30.2) | 37 (69.8) | 1.15 (0.58, 2.34) | 0.689 |  |
| Tumor type |  |  |  |  |  |
| *Adenocarcinoma, large cell or undifferentiated* | 61 (24.3) | 190 (75.7) | Ref. | Ref. |  |
| *Squamous cell carcinoma* | 26 (24.5) | 80 (75.5) | 0.99 (0.58, 1.69) | 0.957 |  |
| Smoking habits |  |  |  |  |  |
| *Current smoker* | 43 (29.9) | 101 (70.1) | Ref. | Ref. |  |
| *Former smoker* | 38 (22.1) | 134 (77.9) | 1.50 (0.90, 2.50) | 0.119 |  |
| *Non-smoker* | 6 (15.8) | 32 (84.2) | 2.22 (0.91, 6.33) | 0.080 |  |
| Stage of cancer |  |  |  |  |  |
| *IA* | 10 (23.8) | 32 (76.2) | Ref. | Ref. |  |
| *IB* | 9 (18.4) | 40 (81.6) | 1.38 (0.49, 3.93) | 0.537 |  |
| *IIA* | 3 (30.0) | 7 (70.0) | 0.72 (0.16, 4.07) | 0.687 |  |
| *IIB* | 5 (26.3) | 14 (73.7) | 0.87 (0.25, 3.31) | 0.828 |  |
| *IIIA* | 8 (29.6) | 19 (70.4) | 0.74 (0.25, 2.30) | 0.601 |  |
| *IIIB-C* | 8 (17.8) | 37 (82.2) | 1.43 (0.50, 4.25) | 0.503 |  |
| *IV* | 45 (27.6) | 118 (72.4) | 0.83 (0.36, 1.78) | 0.637 |  |
| Categorical variables are described with frequencies and percentages. CI, confidence interval; CNB, core needle biopsy; EBUS, endobronchial ultrasound; OR, odds-ratio; Ref., reference level. Missing values: sample collection procedure (n=3 in non-informative, n=9 in informative); tumor type (n=3 in non-informative, n=5 in informative); smoking habits (n=3 in non-informative, n=8 in informative); stage of cancer (n=2 in non-informative, n=8 in informative). | | | | | |

**Table S4**. Metrics results

| **Measure** | **All tumors**  **N = 404** | **Lung tumors**  **N = 275** |
| --- | --- | --- |
| DNA mass, ng | 27.9 (21.4,42.9) | 25.6 (20.8,39.0) |
| Sample quality according to DNA mass |  |  |
| *Good quality* | 396 (98.0) | 268 (97.5) |
| *Bad quality* | 8 (1.98) | 7 (2.55) |
| Q-ratio | 0.55 (0.30,0.86) | 0.63 (0.34,0.91) |
| Sample quality according to Q-ratio |  |  |
| *Good quality* | 403 (99.8) | 275 (100) |
| *Bad quality* | 1 (0.25) | 0 (0.0) |
| Number of reading pairs | 18791310  (15486185, 23202832) | 18602243  (15444236, 22899625) |
| Median sequencing depth | 9285 (7059, 13125) | 10054 (7362, 13768) |
| 5^th^ percentile of sequencing depth | 2868 (1988, 4425) | 3102 (2092, 5096) |
| 95^th^ percentile of sequencing depth | 19897 (16712, 24748) | 20239 (16888, 25266) |
| Median unique depth | 1886 (1306, 2414) | 1950 (1282, 2444) |
| 5^th^ percentile of unique depth | 784 (480, 1121) | 852 (496, 1236) |
| 5^th^ percentile of unique depth over 500 |  |  |
| *≥500* | 295 (73.0) | 204 (74.2) |
| *<500* | 109 (27.0) | 71 (25.8) |
| 95^th^ percentile of unique depth | 2991 (2165, 3772) | 2985 (2042, 3808) |
| Median fragment length | 148 (134, 161) | 150 (137, 164) |
| 5^th^ percentile of fragment length | 102 (101, 103) | 102 (101, 104) |
| 95^th^ percentile of fragment length | 276 (230, 315) | 286 (240, 322) |
| Categorical measures are described with frequencies and percentages. For quantitative measures, median and percentiles of 25% and 75% are shown. | | |

**Table S5**. Population prevalence of genomic alterations for all tumors

| **Gen** | **n** | **Prevalence (95% CI)^a^** |
| --- | --- | --- |
| TP53 | 208 | 51.5 (46.5, 56.5) |
| KRAS | 118 | 29.2 (24.8, 33.9) |
| APC | 70 | 17.3 (13.8, 21.4) |
| EGFR | 42 | 10.4 (7.60, 13.8) |
| BRAF | 35 | 8.70 (6.10, 11.8) |
| ALK | 23 | 5.70 (3.60, 8.40) |
| KIT | 21 | 5.20 (3.20, 7.80) |
| PIK3CA | 20 | 5.00 (3.00, 7.50) |
| CDKN2A | 17 | 4.20 (2.50, 6.70) |
| MET | 17 | 4. 20 (2.50, 6.70) |
| ERBB2 | 14 | 3.50 (1.90, 5.70) |
| STK11 | 13 | 3.20 (1.70, 5.40) |
| NRAS | 12 | 3.00 (1.50, 5.10) |
| DPYD | 10 | 2.50 (1.20, 4.50) |
| FGFR2 | 8 | 2.00 (0.86, 3.90) |
| CTNNB1 | 7 | 1.70 (0.70, 3.50) |
| PTEN | 7 | 1.70 (0.70, 3.50) |
| SMAD4 | 7 | 1.70 (0.70, 3.50) |
| FBXW7 | 6 | 1.50 (0.55, 3.20) |
| PDGFRA | 6 | 1.50 (0.55, 3.20) |
| MTOR | 5 | 1.20 (0.40, 2.90) |
| RET | 5 | 1.20 (0.40, 2.90) |
| KEAP1 | 4 | 0.99 (0.27, 2.50) |
| NFE2L2 | 4 | 0.99 (0.27, 2.50) |
| PMS2 | 4 | 0.99 (0.27, 2.50) |
| RB1 | 4 | 0.99 (0.27, 2.50) |
| AR | 3 | 0.74 (0.15, 2.20) |
| KDR | 3 | 0.74 (0.15, 2.20) |
| SMO | 3 | 0.74 (0.15, 2.20) |
| CDK4 | 2 | 0.50 (0.060, 1.80) |
| DDR2 | 2 | 0.50 (0.060, 1.80) |
| FGFR3 | 2 | 0.50 (0.060, 1.80) |
| FLT1 | 2 | 0.50 (0.060, 1.80) |
| FLT4 | 2 | 0.50 (0.060, 1.80) |
| MSH6 | 2 | 0.50 (0.060, 1.80) |
| PIK3R1 | 2 | 0.50 (0.060, 1.80) |
| PTCH1 | 2 | 0.50 (0.060, 1.80) |
| ROS1 | 2 | 0.50 (0.060, 1.80) |
| ABL1 | 1 | 0.25 (0.006, 1.40) |
| AKT1 | 1 | 0.25 (0.006, 1.40) |
| ARAF | 1 | 0.25 (0.006, 1.40) |
| BRCA2 | 1 | 0.25 (0.006, 1.40) |
| CCND1 | 1 | 0.25 (0.006, 1.40) |
| ESR1 | 1 | 0.25 (0.006, 1.40) |
| GATA3 | 1 | 0.25 (0.006, 1.40) |
| GNAS | 1 | 0.25 (0.006, 1.40) |
| KIF5B | 1 | 0.25 (0.006, 1.40) |
| MAP2K2 | 1 | 0.25 (0.006, 1.40) |
| NTRK1 | 1 | 0.25 (0.006, 1.40) |
| PDGFRB | 1 | 0.25 (0.006, 1.40) |
| RAF1 | 1 | 0.25 (0.006, 1.40) |
| a. Prevalence calculated over the total evaluable samples (N=404). | | |

**Table S6**. Population prevalence of genomic alterations for lung tumors

| **Gen** | **n** | **Prevalence (95% CI)^a^** |
| --- | --- | --- |
| TP53 | 162 | 58.9 (52.8, 64.8) |
| KRAS | 75 | 27.3 (22.1, 32.9) |
| EGFR | 37 | 13.5 (9.7, 18.1) |
| ALK | 21 | 7.60 (4.80, 11.4) |
| BRAF | 16 | 5.80 (3.40, 9.30) |
| MET | 15 | 5.50 (3.10, 8.80) |
| CDKN2A | 12 | 4.40 (2.30, 7.50) |
| STK11 | 11 | 4.00 (2.00, 7.00) |
| ERBB2 | 10 | 3.60 (1.80, 6.60) |
| APC | 8 | 2.90 (1.30, 5.70) |
| DPYD | 7 | 2.50 (1.00, 5.20) |
| FGFR2 | 7 | 2.50 (1.00, 5.20) |
| PIK3CA | 7 | 2.50 (1.00, 5.20) |
| KEAP1 | 4 | 1.50 (0.40, 3.70) |
| KIT | 4 | 1.50 (0.40, 3.70) |
| NFE2L2 | 4 | 1.50 (0.40, 3.70) |
| PTEN | 4 | 1.50 (0.40, 3.70) |
| RB1 | 4 | 1.50 (0.40, 3.70) |
| RET | 4 | 1.50 (0.40, 3.70) |
| CTNNB1 | 3 | 1.10 (0.23, 3.20) |
| KDR | 3 | 1.10 (0.23, 3.20) |
| NRAS | 3 | 1.10 (0.23, 3.20) |
| PDGFRA | 3 | 1.10 (0.23, 3.20) |
| CDK4 | 2 | 0.73 (0.088, 2.60) |
| FBXW7 | 2 | 0.73 (0.088, 2.60) |
| FGFR3 | 2 | 0.73 (0.088, 2.60) |
| PMS2 | 2 | 0.73 (0.088, 2.60) |
| ROS1 | 2 | 0.73 (0.088, 2.60) |
| SMO | 2 | 0.73 (0.088, 2.60) |
| ABL1 | 1 | 0.36 (0.009, 2.00) |
| AR | 1 | 0.36 (0.009, 2.00) |
| ARAF | 1 | 0.36 (0.009, 2.00) |
| CCND1 | 1 | 0.36 (0.009, 2.00) |
| DDR2 | 1 | 0.36 (0.009, 2.00) |
| ESR1 | 1 | 0.36 (0.009, 2.00) |
| FLT1 | 1 | 0.36 (0.009, 2.00) |
| KIF5B | 1 | 0.36 (0.009, 2.00) |
| NTRK1 | 1 | 0.36 (0.009, 2.00) |
| PIK3R1 | 1 | 0.36 (0.009, 2.00) |
| RAF1 | 1 | 0.36 (0.009, 2.00) |
| SMAD4 | 1 | 0.36 (0.009, 2.00) |
| a. Prevalence calculated over the total evaluable samples (N=275). | | |

**Table S7**. Principal variants among INDEL and SNV mutations

| **Variant** | **All tumors**  **N = 653** | **Lung tumors**  **N = 386** |
| --- | --- | --- |
| Missense variant | 456 (69.8) | 277 (71.8) |
| Stop gained | 99 (15.2) | 48 (12.4) |
| Frameshift variant | 17 (2.60) | 5 (1.30) |
| Splice acceptor variant & Intron variant | 13 (1.99) | 9 (2.33) |
| Missense variant & Splice region variant | 11 (1.68) | 9 (2.33) |
| Splice donor variant & Intron variant | 10 (1.53) | 8 (2.07) |
| Inframe deletion | 10 (1.53) | 7 (1.81) |
| Disruptive inframe deletion | 8 (1.23) | 7 (1.81) |
| Stop gained & Splice region variant | 8 (1.23) | 2 (0.52) |
| Synonymous variant | 7 (1.07) | 3 (0.78) |
| Splice region variant & Intron variant | 4 (0.61) | 3 (0.78) |
| Splice region variant & Synonymous variant | 4 (0.61) | 3 (0.78) |
| Disruptive inframe insertion | 3 (0.46) | 3 (0.78) |
| Inframe insertion | 1 (0.15) | 1 (0.26) |
| Splice acceptor variant & Splice region variant & Intron variant | 1 (0.15) | 1 (0.26) |
| Frameshift variant & Splice region variant | 1 (0.15) | 0 (0.00) |
| Variants are described with frequencies and percentages. Missing values: n=11 in all tumors, n=4 in lung tumors. | | |

**Table S8**. Sequencing depth measures and number of exons in principal variants by mutational class

| **Measure** | **All tumors** | **Lung tumors** |
| --- | --- | --- |
| Sequencing depth of principal variants |  |  |
| INDEL | 391 (196.2, 671.8) | 358 (163, 616) |
| SNV | 297 (127, 624) | 273 (111, 564.5) |
| Sequencing unique read depth |  |  |
| INDEL | 2112.5 (1545, 2869.5) | 2191 (1520, 2775) |
| SNV | 1635 (1025, 2434) | 1462 (922, 2269.5) |
| Number of exons |  |  |
| INDEL | 16 (16, 19) | 19 (19, 19) |
| SNV | 6 (3, 11) | 6 (3, 8) |
| Total exons |  |  |
| INDEL | 21 (16, 28) | 28 (27, 28) |
| SNV | 11 (7, 16) | 11 (6, 11) |
| Measures are described with median and percentiles of 25% and 75%. Missing values: n=11 in all tumors, n=4 in lung tumors.  INDEL, insertion–deletion; SNV, single nucleotide variant. | | |

**Table S9**. Univariable analysis of lung cancer ESCAT I actionable mutations with reimbursed targeted therapy in Spain

| **Characteristic** | **No**  **N = 238** | **Yes**  **N = 37** | **OR (95% CI)** | **p-value** |
| --- | --- | --- | --- | --- |
| Center |  |  |  |  |
| *1* | 146 (83.0) | 30 (17.0) | Ref. | Ref. |
| *2* | 26 (89.7) | 3 (10.3) | n.e. | - |
| *3* | 33 (100) | 0 (0.00) | n.e. | - |
| *4* | 29 (90.6) | 3 (9.38) | n.e. | - |
| *5* | 4 (80.0) | 1 (20.0) | n.e. | - |
| Sample collection procedure |  |  |  |  |
| *CNB or EBUS-CNB* | 82 (91.1) | 8 (8.89) | Ref. | Ref. |
| *Other diagnostic biopsies* | 55 (84.6) | 10 (15.4) | 1.85 (0.68, 5.21) | 0.228 |
| *Surgical specimen* | 63 (85.1) | 11 (14.9) | 1.77 (0.67, 4.90) | 0.248 |
| *Cytology* | 32 (86.5) | 5 (13.5) | 1.61 (0.44, 5.31) | 0.449 |
| Tumor type |  |  |  |  |
| *Adenocarcinoma, large cell or undifferentiated* | 155 (81.6) | 35 (18.4) | Ref. | Ref. |
| *Squamous cell carcinoma* | 79 (98.8) | 1 (1.25) | 0.06 (0.00, 0.30) | <0.001 |
| Smoking habits |  |  |  |  |
| *Current smoker* | 100 (99.0) | 1 (0.99) | Ref. | Ref. |
| *Former smoker* | 116 (86.6) | 18 (13.4) | n.e. | - |
| *Non-smoker* | 15 (46.9) | 17 (53.1) | n.e. | - |
| Smoking habits |  |  |  |  |
| *Current / Former smoker* | 216 (91.9) | 19 (8.09) | Ref. | Ref. |
| *Non-smoker* | 15 (46.9) | 17 (53.1) | 12.6 (5.47, 29.9) | <0.001 |
| Stage |  |  |  |  |
| *IA* | 28 (87.5) | 4 (12.5) | Ref. | Ref. |
| *IB* | 31 (77.5) | 9 (22.5) | n.e. | - |
| *IIA* | 6 (85.7) | 1 (14.3) | n.e. | - |
| *IIB* | 14 (100) | 0 (0.00) | n.e. | - |
| *IIIA* | 18 (94.7) | 1 (5.26) | n.e. | - |
| *IIIB-C* | 33 (89.2) | 4 (10.8) | n.e. | - |
| *IV* | 101 (85.6) | 17 (14.4) | n.e. | - |
| PD-L1 expression categories |  |  |  |  |
| *Negative [0, 1)* | 85 (88.5) | 11 (11.5) | Ref. | Ref. |
| *Positive non-overexpressed [1, 50)* | 65 (80.2) | 16 (19.8) | 1.89 (0.82, 4.49) | 0.135 |
| *Positive overexpressed ≥50* | 82 (90.1) | 9 (9.89) | 0.85 (0.32, 2.19) | 0.738 |
| Categorical variables are described with frequencies and percentages. For PD-L1 level, median and percentiles of 25% and 75% are shown. CI, confidence interval; CNB, core needle biopsy; EBUS, endobronchial ultrasound; OR, odds-ratio; Ref., reference level; n.e, not estimable. Missing values: sample collection procedure (n=6 in no, n=3 in yes); tumor type (n=4 in no, n=1 in yes); smoking habits (n=7 in no, n=1 in yes); stage of cancer (n=7 in no, n=1 in yes); PD-L1 level (n=6 in no, n=1 in yes). | | | | |

**Table S10**. Univariable analysis of lung cancer actionable mutations ESCAT I-II

| **Characteristic** | **No**  **N = 202** | **Yes**  **N = 73** | **OR (95% CI)** | **p-value** |
| --- | --- | --- | --- | --- |
| Center |  |  |  |  |
| *1* | 124 (70.5) | 52 (29.5) | Ref. | Ref. |
| *2* | 24 (82.8) | 5 (17.2) | 0.51 (0.16, 1.32) | 0.174 |
| *3* | 27 (81.8) | 6 (18.2) | 0.54 (0.19, 1.32) | 0.184 |
| *4* | 24 (75.0) | 8 (25.0) | 0.80 (0.32, 1.85) | 0.621 |
| *5* | 3 (60.0) | 2 (40.0) | 1.62 (0.18, 10.9) | 0.627 |
| Sample collection procedure |  |  |  |  |
| *CNB or EBUS-CNB* | 69 (76.7) | 21 (23.3) | Ref. | Ref. |
| *Other biopsies* | 47 (72.3) | 18 (27.7) | 1.26 (0.60, 2.63) | 0.543 |
| *Surgical specimen* | 57 (77.0) | 17 (23.0) | 0.98 (0.47, 2.04) | 0.960 |
| *Cytology* | 24 (64.9) | 13 (35.1) | 1.77 (0.76, 4.10) | 0.185 |
| Tumor type |  |  |  |  |
| *Adenocarcinoma, large cell or undifferentiated* | 125 (65.8) | 65 (34.2) | Ref. | Ref. |
| *Squamous cell carcinoma* | 73 (91.2) | 7 (8.75) | 0.19 (0.07, 0.41) | <0.001 |
| Smoking habits |  |  |  |  |
| *Current smoker* | 85 (84.2) | 16 (15.8) | Ref. | Ref. |
| *Former smoker* | 100 (74.6) | 34 (25.4) | 1.79 (0.94, 3.56) | 0.079 |
| *Non-smoker* | 12 (37.5) | 20 (62.5) | 8.61 (3.57, 21.8) | <0.001 |
| Smoking habits |  |  |  |  |
| *Current / Former smoker* | 185 (78.7) | 50 (21.3) | Ref. | Ref. |
| *Non-smoker* | 12 (37.5) | 20 (62.5) | 6.08 (2.81, 13.7) | <0.001 |
| Stage |  |  |  |  |
| *IA* | 26 (81.2) | 6 (18.8) | Ref. | Ref. |
| *IB* | 30 (75.0) | 10 (25.0) | 1.43 (0.46, 4.80) | 0.546 |
| *IIA* | 3 (42.9) | 4 (57.1) | 5.38 (0.91, 36.6) | 0.064 |
| *IIB* | 10 (71.4) | 4 (28.6) | 1.72 (0.36, 7.66) | 0.482 |
| *IIIA* | 16 (84.2) | 3 (15.8) | 0.83 (0.15, 3.77) | 0.817 |
| *IIIB-C* | 29 (78.4) | 8 (21.6) | 1.19 (0.36, 4.14) | 0.781 |
| *IV* | 82 (69.5) | 36 (30.5) | 1.86 (0.74, 5.44) | 0.195 |
| PD-L1 expression categories |  |  |  |  |
| *Negative [0, 1)* | 75 (78.1) | 21 (21.9) | Ref. | Ref. |
| *Positive non-overexpressed [1, 50)* | 55 (67.9) | 26 (32.1) | 1.68 (0.86, 3.33) | 0.131 |
| *Positive overexpressed ≥50* | 67 (73.6) | 24 (26.4) | 1.28 (0.65, 2.53) | 0.478 |
| Categorical variables are described with frequencies and percentages. For PD-L1 level, median and percentiles of 25% and 75% are shown. CI, confidence interval; CNB, core needle biopsy; EBUS, endobronchial ultrasound ;OR, odds-ratio; Ref., reference level. Missing values: sample collection procedure (n=4 in no, n=5 in yes); tumor type (n=4 in no, n=1 in yes); smoking habits (n=4 in no, n=4 in yes); stage of cancer (n=5 in no, n=3 in yes); PD-L1 level (n=5 in no, n=2 in yes). | | | | |
